# Comment on “Differences in Ventilatory Threshold for Exercise Prescription in Outpatient Diabetic and Sarcopenic Obese Subjects”

**DOI:** 10.1155/2017/1754215

**Published:** 2017-11-16

**Authors:** Goran Kuvačić, Johnny Padulo

**Affiliations:** ^1^Faculty of Kinesiology, University of Split, Split, Croatia; ^2^University eCampus, Novedrate, Italy; ^3^Research Laboratory “Sport Performance Optimization”, National Center of Medicine and Sciences in Sport (CNMSS), Tunis, Tunisia

Recently, we read in *International Journal of Endocrinology* an article entitled “Differences in Ventilatory Threshold for Exercise Prescription in Outpatient Diabetic and Sarcopenic Obese Subjects” on exercise intensity prescription in diabetic, sarcopenic, and control obese subjects [1]. We think that this article promotes a convincing approach, but one worthy of stronger methodological support. Some reflections in this letter point out what is yet necessary to authoritatively suggest the effectiveness of this approach.

In particular, the methodological approach shows some severe flaws, which might undermine the interpretation of the results. Therefore, this letter aims to suggest improvements to achieve stronger conclusions to this study.

In the methods, “Clinical Evaluation” it is stated that “All subjects were evaluated in the morning” without providing any further information for circadian effects (time of day) and seasonal period [2] control. To evaluate body composition, the authors use both DEXA and BIA without justifying this choice, that is, Why both? Why not only one of them? If so, which one? However, they neglect to write that BIA has shown a 5% error [3], which might have reduced the result's accuracy. And if that were the case, the risk of “signal” to be corrupted by “noise” would be too high for a reliable scientific study [4]. In “2.2. Clinical Evaluation” it is noted that “A low-calorie diet was set at approximately 400 Kcal less than total daily energy expenditure in all patients.” A calorie income decrease, set very similar for all the subjects, that is, not body mass-individualised, has at least to be further clarified given the documented body mass differences; for example, in sarcopenic obese subjects, body mass percent coefficient of variation amounts to 25%.

More information about the “Pulmonary function tests and a resting electrocardiogram” outcome is needed, given that some basic results are not presented. Such information is essential to allow other scientists to replicate the authors' approach.

In “2.3. Maximal Effort and Individual Ventilatory Threshold,” the authors support the use of the chosen treadmill incremental protocol by means of a self-citation, which might be proper only if the provided self-citation would really describe the protocol. Yet, this is not the case, because the self-citation refers only to an undescribed “modified Balke protocol.” Therefore, the authors should describe the chosen treadmill incremental protocol and provide an original reference (the one used in the self-citation, [27] B. Balke and R. W. Ware, “An experimental study of physical fitness of Air Force personnel,” *United States Armed Forces Medical Journal*, vol. 10, no. 6, pp. 675–688, 1959, would fit well). Furthermore, the authors do not provide any information on correct treadmill use for scientific research, for example, on speed calibration or slope setting [5].

In “Maximal Effort and Individual Ventilatory Threshold,” the authors support the use of the RPE scale by means of a reference validating its use in 18–36-year-old people, while the article's study participants are ~60 years old [6]. Finally, the authors use one-way ANOVA without disclosing *F* values, in-so-doing preventing the reader from verifying the study's power. No matter what may have happened over the submission and revision process, we strongly believe such statistical information must be provided, because it is the main way to allow the readers to check statistical relevance.

In conclusion, we believe that—especially for strengthening the study's conclusions—the article needs deep revising about the above-explained research method issues. Diabetic patients' healthcare is really worthy of attention by the scientific community, but scientists have to provide useful practical indications obtained only by means of sound methodological support.
